# Application of AI-Generated Content in Medical Education: Systematic Review of the Impact on Critical Thinking Abilities of Medical Students

**DOI:** 10.2196/79939

**Published:** 2026-02-27

**Authors:** Jinlei Li, Fen Ai, Jueyan Wang, Bingxin Cheng, Yu Li, Zhen Chen

**Affiliations:** 1 School of Medicine, Jianghan University, Wuhan, China; 2 Department of Emergency Medicine, The Central Hospital of Wuhan, Tongji Medical College, Huazhong University of Science and Technology, 26 Shengli Street, Jiang’an District, Wuhan, 430000, China, 86 15673077558

**Keywords:** artificial intelligence–generated content, AIGC, medical students, large language models, LLM, ChatGPT, critical thinking

## Abstract

**Background:**

With the rapid development of artificial intelligence technology, artificial intelligence–generated content (AIGC) is increasingly widely applied in the field of medical education. Large language models, such as ChatGPT, are a prominent type of AIGC technology. Critical thinking is a core ability in medical education, but the impact of AIGC technology on the critical thinking ability of medical students remains unclear. Medical students are at a crucial stage in cultivating critical thinking, and the intervention of AIGC technology may have a profound impact on this process.

**Objective:**

This study aims to systematically review the impact of AIGC technology on the complex mechanisms affecting medical students’ critical thinking abilities and build a corresponding strategic framework. The findings are intended to provide theoretical support and practical guidance for applying AIGC in medical education.

**Methods:**

This study followed 2020 PRISMA (Preferred Reporting Items for Systematic Reviews and Meta-Analyses) guidelines, with the retrieval scope limited to English studies published between November 2022 and June 2025. Through the PubMed database, combined with the search methods of subject terms and free words, relevant studies involving the impact of AIGC on the critical thinking of medical students were screened for using keywords such as “AIGC,” “medical students,” and “critical thinking.” Two independent reviewers screened and evaluated the literature, and ultimately conducted a qualitative analysis based on the common themes extracted from the literature.

**Results:**

AIGC technology in medical education is 2-fold. First, AIGC’s powerful information capabilities provide abundant learning resources and efficient tools. This accelerates knowledge acquisition and broadens learning scope. Second, overreliance on AIGC may lead to mental inertia, weaken critical thinking skills, and cause academic integrity issues among students. Research has found that strategies such as customized AIGC tools, virtual standardized patients, new models of resource integration, and proactive assessment of AI limitations can effectively make up for the deficiencies of AIGC in cultivating high-level critical thinking, helping medical students maintain and enhance their critical thinking and problem-solving abilities.

**Conclusions:**

AIGC technology application in medical education needs to carefully weigh the pros and cons. By optimizing the design and usage of AIGC tools and combining them with the guidance and supervision of educators, they can be transformed into powerful tools for promoting the development of critical thinking among medical students. Future research should further expand the scope of study, optimize research methods, pay attention to individual differences, track long-term effects, and deeply explore the influence of ethical and cultural factors to more comprehensively assess the application potential and challenges of AIGC technology in medical education.

## Introduction

Critical thinking is defined as a deliberate and self-regulatory cognitive process that involves the examination and evaluation of information to arrive at evidence-based conclusions. Within the medical domain, critical thinking is primarily manifested in 4 key areas: medical decision-making, ethical considerations, evidence-based medical practice, and scientific research capabilities. In the context of medical decision-making, health care professionals are required to synthesize multidimensional evidence, develop comprehensive diagnostic and treatment strategies, and ensure that their decisions are both scientifically sound and effective. From an ethical and safety standpoint, practitioners must recognize and rectify cognitive biases, avoid logical fallacies, and uphold standards of medical safety and ethical integrity. In the realm of evidence-based medicine, physicians are tasked with critically appraising research findings and discerning high-quality evidence to enhance clinical decision-making. Regarding scientific research competencies, medical professionals must possess strong logical reasoning, hypothesis testing skills, data analysis capabilities, and the ability to interpret results, all of which are essential for advancing medical research and clinical practice [1,2].

For medical students, the development of critical thinking skills is of paramount importance. This group is at a pivotal juncture in their journey to becoming physicians. This period represents an optimal time for fostering critical thinking abilities. Students who lack these skills may become passive recipients of knowledge. They will likely struggle to navigate the complexities, uncertainties, and unpredictabilities inherent in clinical practice [3]. Consequently, medical education should prioritize the cultivation of critical thinking, equipping students with the skills necessary to actively engage in problem-solving, analysis, and critical evaluation, thereby establishing a robust foundation for their future careers in medicine.

Artificial intelligence (AI)–generated content (AIGC) encompasses the application of generative AI technology to autonomously produce multimodal content, including text, images, and audio. A prominent category of AIGC tools is large language models (LLMs), which are designed to understand and generate humanlike text. ChatGPT is a widely used example of an LLM. By the year 2025, medical schools across the globe have progressively incorporated AIGC tools into their curricula. For instance, ChatGPT has demonstrated performance comparable to that of human candidates on the United States Medical Licensing Examination [4]. While AIGC can enhance educational efficiency, it raises significant concerns about its impact on critical thinking skills. Seetharaman et al [5] note that contemporary medical education often overemphasizes objective assessments while neglecting subjective competencies, such as communication and critical thinking. In this context, although AIGC tools (eg, LLMs such as ChatGPT) can simulate doctor-patient interactions, their use risks undermining the development of critical thinking in medical students.

**Figure 1.**
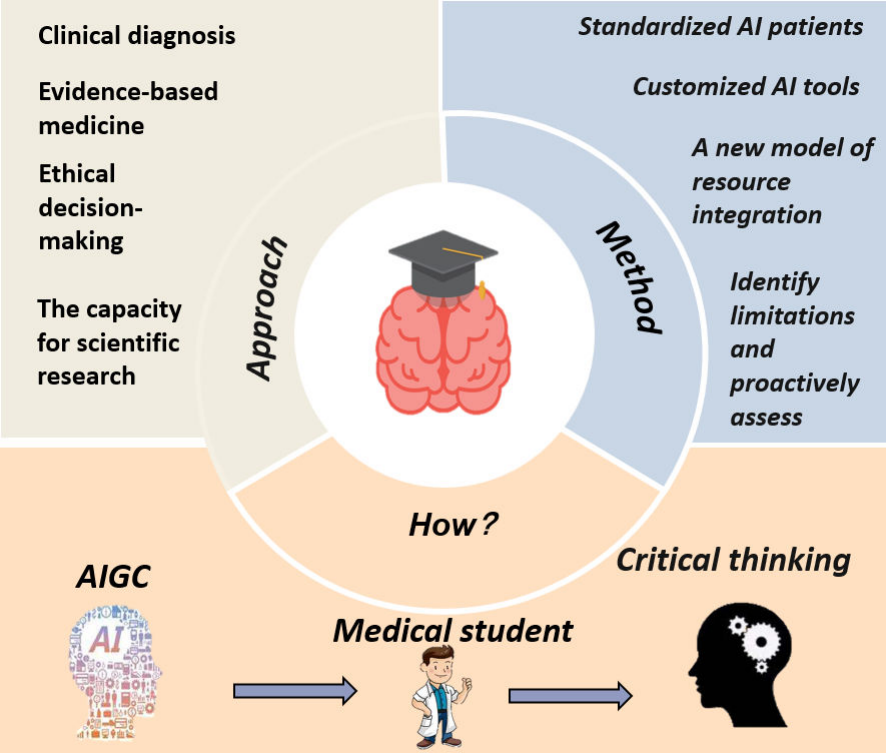
Complex mechanism of artificial intelligence (AI)–generated content (AIGC) on the critical thinking ability of medical students.

**Figure 2.**
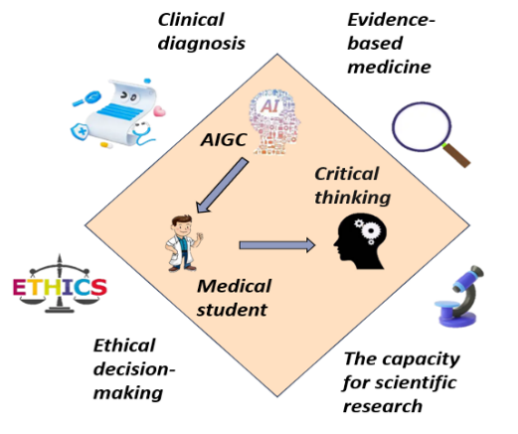
Application of artificial intelligence (AI)–generated content (AIGC) to the critical thinking of medical students.

This study seeks to elucidate the intricate mechanisms through which AIGC technology influences the critical thinking abilities of medical students, using a systematic literature review methodology. As illustrated in Figure 1, our proposed framework delineates a dual-pathway model wherein AIGC both supports and potentially hinders critical thinking development through various cognitive and educational mediators. The investigation encompasses a comprehensive analysis of global empirical studies on AIGC, with a particular focus on tools such as ChatGPT, from its public release in November 2022 through June 2025. Notably, this research introduces the novel “dual-path action model” and concentrates on 4 fundamental dimensions: clinical diagnostic reasoning, evidence-based medical practice, ethical decision-making, and scientific research innovation. Figure 2 provides a conceptual map of how AIGC applications map onto these 4 critical thinking dimensions, offering a visual summary of our analytical approach. By thoroughly examining the differentiated effects of AIGC applications on critical thinking within each dimension, the study aims to establish a robust theoretical foundation to inform targeted interventions and guide the development of educational policies.

In light of the pivotal transition occurring in medical education, this study seeks to address a fundamental question: how can the critical thinking skills of medical students be safeguarded and enhanced while simultaneously leveraging the capabilities of AIGC technology? The findings of this inquiry will have significant implications for the quality of future medical professionals and will also influence the preservation of the humanistic aspects of medicine amid the ongoing technological revolution.

## Methods

### PRISMA Flowchart-Based Literature Screening and Analysis

This systematic review aims to methodically identify, evaluate, and synthesize existing literature to elucidate the specific effects of AIGC on the critical thinking abilities of medical students. The review was conducted in strict accordance with the PRISMA (Preferred Reporting Items for Systematic Reviews and Meta-Analyses) 2020 guidelines and used a systematic methodology to assess the influence of AIGC on medical students’ critical thinking (Checklist 1). Additionally, the review protocol was registered with PROSPERO under the registration number CRD420251116204.

### Retrieval Strategy and Information Sources

This study used a combined search strategy utilizing both controlled vocabulary (subject terms) and free-text terms (refer to Table 1 for specifics) to perform a comprehensive literature search within the PubMed database. The search focused on keywords including AIGC and its prominent applications such as LLMs, medical students, and critical thinking. To ensure the inclusion of the most current and relevant research, the search was restricted to studies published subsequent to the introduction of ChatGPT, thereby capturing the latest advancements in this domain.

**Table 1.** PubMed search strategy for identifying studies.

Search	Query
1	(“Medical Student” [All Fields] OR “Medical Students” [All Fields] OR “Student, Medical”[All Fields])
2	(“Generative Artificial Intelligence” [All Fields] OR “Artificial Intelligence, Generative” [All Fields] OR “Chatbot” [All Fields] OR “Chatbots” [All Fields] OR “Chat-GPT” [All Fields] OR “Chat GPT” [All Fields] OR “ChatGPT” [All Fields] OR “ChatGPTs” [All Fields])
3	(Critical Thinking OR Thinking, Critical OR Thinking Skills OR Thinking Skill OR Thought OR Thoughts)
4	1 AND 2 AND 3
5	4 Limits:y_5[Filter]

### Research Screening and Inclusion and Exclusion Criteria

The inclusion criteria are relevant studies involving the impact of AIGC on the critical thinking of medical students. The exclusion criterion is that the full text of the literature cannot be obtained or it is not published in English.

### Risk of Bias Assessment and Data Extraction

Considering the variations in research design types, this study used a tiered quality assessment approach. Specifically, the Cochrane RoB 2.0 tool was used to assess the risk of bias in randomized controlled trials. For nonrandomized intervention studies, the ROBINS-I framework was applied to conduct a systematic evaluation. For other study designs, including observational, qualitative, or mixed methods research, the JBI series of checklists—endorsed by international consensus and tailored to specific design types—was selected to ensure methodological rigor and comparability of evidence quality.

### The Main Results of Literature Retrieval

Two reviewers (JW and ZC) independently screened the literature. First, they performed a preliminary screening of titles and abstracts to exclude obviously irrelevant studies. Then, they conducted a full-text review of the selected studies. This review focused on the relevance of AIGC to the development of critical thinking skills in medical students. Throughout the review, a comprehensive appraisal was conducted regarding the methodological rigor, the reliability of the findings, and the validity of the conclusions presented. Key themes pertaining to the application of AIGC in enhancing critical thinking among medical students were identified and extracted. In cases of disagreement between the 2 reviewers during the screening process, consensus was achieved through detailed discussion to maintain the objectivity and accuracy of the selection outcomes. A qualitative analysis was subsequently performed based on the recurring themes identified in the literature, with the objective of providing a robust and evidence-based foundation to inform the policy development and practical implementation of AI technologies within the academic medical domain.

## Results

### Overview

**Figure 3.**
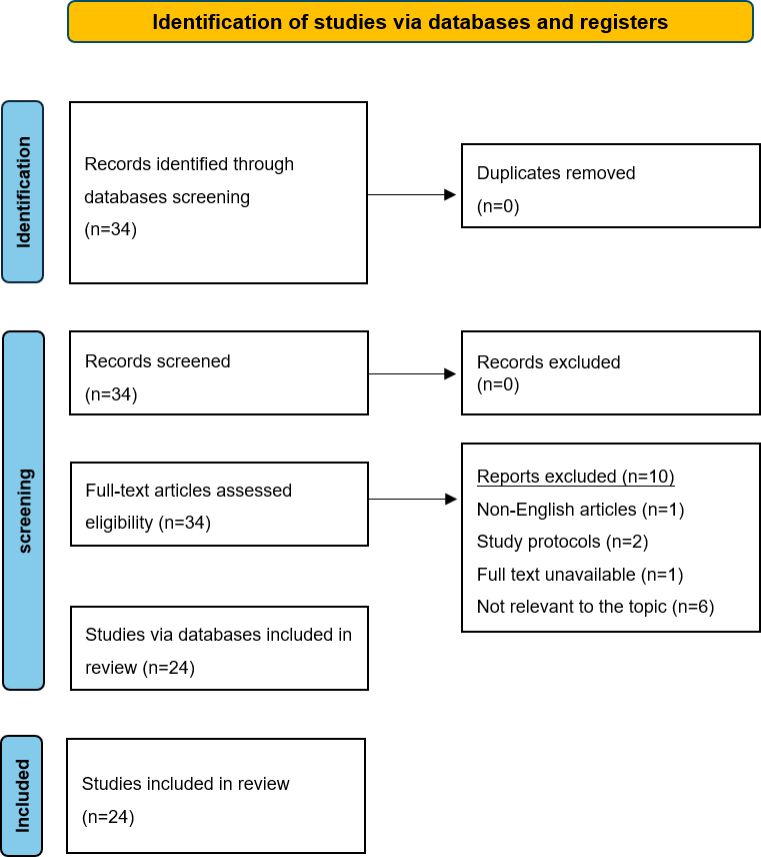
Flowchart of the system review. This flowchart shows the screening process of the included and excluded studies and marks the reasons for exclusion.

The literature selection process is summarized in the PRISMA flow diagram (Figure 3). The initial database search yielded 34 records. After removing 0 duplicates, 34 records underwent title and abstract screening. Following this, 34 full-text papers were assessed for eligibility, resulting in the final inclusion of 24 studies that met all criteria. The primary reasons for exclusion at the full-text stage were that the studies did not focus on medical students or lacked empirical data on critical thinking.

Table 2 shows the risk assessment and quality appraisal of the included studies, and Table 3 shows the key takeaways.

**Table 2.** Quality appraisal and risk of bias summary of the included studies.

Author and year	Type of research	Evaluation tool	Quality assessment by author JW	Quality assessment by author ZC	Rank
Ahmad et al [6] (2024)	Narrative review	SANRA^a^	6/12	7/12	Moderate quality
Jeyaraman et al [7] (2023)	Opinion review	SANRA	9/12	10/12	High quality
Prober and Desai [8] (2023)	Commentary	SANRA	10/12	9/12	High quality
Magalhães Araujo et al [9] (2024)	Mixed methods study	MMAT^b^	6/7	6/7	High quality
Xu et al [10] (2024)	Review article	SANRA	9/12	10/12	High quality
Madrid et al [11] (2025)	Nonrandomized interventional study	ROBINS-I^c^	Moderate risk	Moderate risk	Moderate quality
Kaliterna et al [12] (2024)	Cross-sectional studies	JBI	6/8	6/8	High quality
Abdullah et al [13] (2024)	Cross-sectional studies	JBI	6/8	5/8	Moderate quality
Moskovic and Rozani [14] (2025)	Mixed methods study	MMAT	6/7	6/7	High quality
Almazrou et al [15] (2024)	Cross-sectional studies	JBI	6/8	7/8	High quality
Abouammoh et al [16] (2025)	Qualitative study	JBI	8/10	8/10	High quality
Jiang et al [17] (2024)	RCT^d^	Cochrane RoB 1.0	Low risk	Low risk	High quality
Öncü et al [18] (2025)	Nonrandomized interventional study	ROBINS-I	High risk	High risk	Low quality
Shalong et al [19] (2024)	RCT	Cochrane RoB^e^ 1.0	Low risk	Low risk	High quality
Reinke et al [20] (2025)	Observational study	JBI	Moderate quality	Moderate quality	Moderate quality
Elabd et al [21] (2025)	Tutorial	JBI	High quality	High quality	High quality
Wang et al [22] (2025)	Qualitative study	JBI	9/10	9/10	High quality
Gong et al [23] (2024)	Nonrandomized interventional study	ROBINS-I	Moderate risk	Moderate risk	Moderate risk
Montagna et al [24] (2025)	RCT	Cochrane RoB-1	Moderate risk	Moderate risk	Moderate risk
Mondal [25] (2025)	Cross-sectional studies	JBI	Moderate quality	Moderate quality	Moderate quality
Laupichler et al [26] (2024)	Nonrandomized interventional study	ROBINS-I	Moderate risk	Moderate risk	Moderate risk
Thomae et al [27] (2024)	Cross-sectional studies	JBI	Moderate quality	Moderate quality	Moderate quality
Mondal [28] (2025)	Expert opinion	JBI	High quality	High quality	High quality
Wu et al [29] (2024)	Expert opinion	JBI	High quality	High quality	High quality

a SANRA: Scale for the Assessment of Narrative Review Articles.

b MMAT: Mixed Methods Appraisal Tool.

c ROBINS-I: Risk of Bias in Non-Randomized Studies of Interventions.

d RCT: randomized controlled trial.

e RoB: Risk of Bias.

**Table 3.** Key takeaways of core studies.

Author and year	Key takeaway
Ahmad et al [6] (2024)	The dual impact of AI^a^ on medical education and clinical practice was explored, with particular attention to potential risks in cognitive development of medical students.
Jeyaraman et al [7] (2023)	The potential and risk of ChatGPT in medicine, education, and scientific research are systematically reviewed, and the core viewpoint of “complementarity rather than substitution” is put forward.
Prober and Desai [8] (2023)	In view of the serious shortage of doctors in the United States, 3 major reform directions are proposed. Appeal to pay attention to the cultivation of critical thinking.
Magalhães Araujo et al [9] (2024)	Application of ChatGPT in medical informatics education.
Xu et al [10] (2024)	The potential impact of AI, especially generative AI (such as GPT-4), on medical education, residency training, and continuing education is discussed.
Madrid et al [11] (2025)	The application potential of LLM^b^ in medical field is discussed, especially the performance of GPT series model in German medical license examination.
Kaliterna et al [12] (2024)	A study on AI’s ability to generate ethical dilemma essays: comparing students’ writing with AI-generated personal ethical dilemma reflection essays.
Abdullah et al [13] (2024)	Investigating ethical issues of ChatGPT in medical education.
Moskovic and Rozani [14] (2025)	The hybrid approach reveals students’ real needs and fears for AI, beyond theoretical speculation.
Almazrou et al [15] (2024)	A cross-sectional survey of medical students’ perceptions of ChatGPT on critical thinking skills.
Abouammoh et al [16] (2025)	This paper discusses the early cognition, experience, and potential challenge of ChatGPT among middle school teachers and students in medical education.
Jiang et al [17] (2024)	A randomized controlled trial was designed to explore the effect of chatbot-based standardized patient combined with case-based learning in colorectal cancer teaching.
Öncü et al [18] (2025)	This paper discusses the application of ChatGPT-4o as virtual standardized patient (SP) in medical education, focusing on the evaluation of clinical case management ability of interns.
Shalong et al [19] (2024)	Randomized controlled trial to assess the impact of LearnGuide on medical students to support autonomous learning among medical students.
Reinke et al [20] (2025)	The integration path of ChatGPT in medical informatics education was explored.
Elabd et al [21] (2025)	Using AI tools to generate the potential value of personalized multimodal memory in medical education, this paper explores the integration path of ChatGPT in medical informatics education.
Wang et al [22] (2025)	This paper discusses the willingness of Chinese medical students to use ChatGPT in programming courses and its influencing factors.
Gong et al [23] (2024)	The potential application of 2 large language models in gastroenterology was discussed.
Montagna et al [24] (2025)	Comparison of 3 types of clinical decision support systems in medical student case solving.
Mondal [25] (2025)	To investigate the use of question-answering resources by medical students in class. To compare the performance of 3 types of clinical decision support systems in medical students’ case solving.
Laupichler et al [26] (2024)	Double-blind control design to compare the quality difference between ChatGPT-3.5 generated questions and artificial questions.
Thomae et al [27] (2024)	Three different modes of integration of ChatGPT in medical curricula were systematically explored, and a comprehensive hybrid approach assessment was conducted.
Mondal [28] (2025)	This paper discusses the opportunities and challenges of large language models (such as ChatGPT) in medical education and proposes a specific ethical framework for their use.
Wu et al [29] (2024)	The potential, application scenarios, and challenges of ChatGPT in medical education were discussed.

a AI: artificial intelligence.

b LLM: large language model.

### Dual Impact of AIGC on Medical Students’ Critical Thinking

By conducting a systematic literature review and synthesis, this study identifies a dual impact of AIGC on the critical thinking abilities of medical students. The subsequent analysis will comprehensively examine both the positive and negative effects of AIGC on medical students’ critical thinking, with particular emphasis on 4 key dimensions: clinical diagnostic reasoning, evidence-based medical practice, ethical decision-making, and scientific research innovation. The objective is to offer theoretical insights and a practical framework to enhance the effective utilization of AIGC tools by medical students.

### AIGC to the Critical Thinking Skills of Medical Students

#### Phenomenon of AI Syndrome

Ahmad et al [6] introduced the notion of “AI syndrome,” cautioning that reliance on AIGC may result in “progressive cognitive decline” among medical students. The authors argue that students tend to favor the immediate responses offered by AIGC, which diminishes their capacity for independent thought and undermines the development of critical clinical reasoning skills. While these short-term efficiencies may be appealing, they could ultimately contribute to a deterioration of cognitive abilities, thereby jeopardizing clinical judgment and patient safety in the long run.

#### Mental Inertia

In 2023, Jeyaraman et al [7] highlighted that while AIGC demonstrates proficiency in memory-related tasks, it exhibits significant shortcomings in deep reasoning, challenging established knowledge and mitigating unwarranted self-confidence. These deficiencies may inadvertently promote a lack of diligence among students, leading them to neglect critical thinking. Concurrently, Prober and Desai [8] advocated for a transformation in medical education, suggesting a shift from prioritizing students with strong memorization skills to fostering essential competencies, such as critical thinking and empathy. This recommendation is underscored by the fact that AI has successfully passed the United States Medical Licensing Examination, indicating a diminishing emphasis on rote memorization. Subsequently, a mixed methods study conducted by Magalhães Araujo et al [9] in 2024 revealed that approximately 20% of students exhibited signs of cognitive inertia, relying heavily on cue words, thereby underscoring the potential adverse effects of AIGC on students’ independent thinking capabilities. In the same year, Xu et al [10] explicitly cautioned against the dangers of excessive dependence on AIGC, warning that it could foster “intellectual laziness.” They noted that the authoritative presentation of information generated by AIGC might lead students to accept such information uncritically, thereby undermining their critical thinking skills. In 2025, Madrid et al [11] further corroborated the limitations of AIGC regarding deep reasoning and the challenge of knowledge in their analysis of the German medical licensing examinations, emphasizing its detrimental effects on students’ cognitive engagement.

#### Academic Integrity

In 2023, Jeyaraman et al [7] were pioneers in identifying the potential challenges associated with AIGC within the realm of medical education, advocating for the creation of detection tools to mitigate the risks of AIGC misuse. Following this, a cross-sectional study conducted by Kaliterna et al [12] in 2024 further elucidated the gravity of the issue, revealing that as many as 34% of reflective papers authored by medical students were likely generated or altered by AI. This occurrence not only underscores the significant threat posed to academic integrity by the misuse of AIGC but also indicates a potential detrimental impact on students’ independent thinking and writing skills.

**Figure 4.**
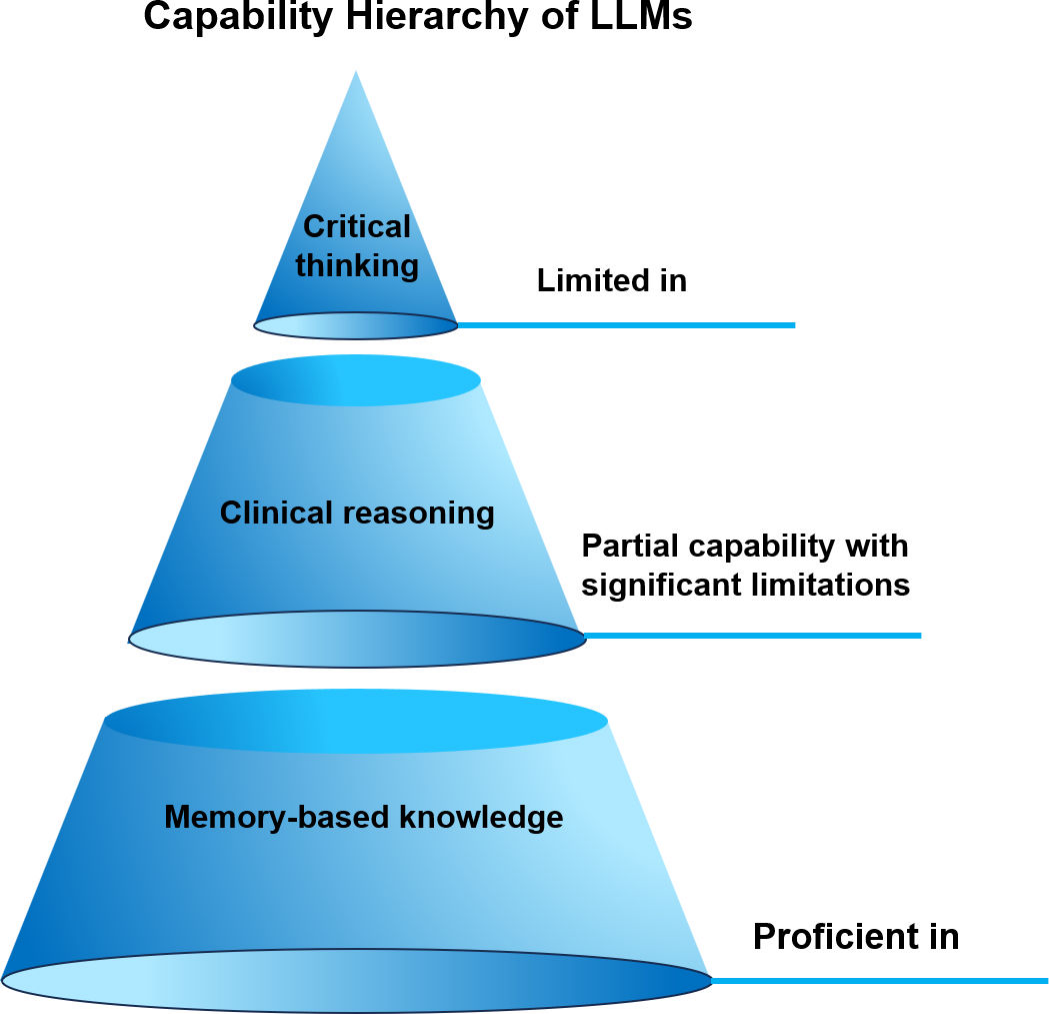
Capability hierarchy of artificial intelligence–generated content (AIGC). LLM: large language model.

These investigations illustrate that while AIGC offers notable advantages in the retrieval and application of memory-based knowledge, thereby providing efficient and convenient support for medical education, it also presents considerable limitations in fostering higher-order critical thinking. As visualized in Figure 4, AIGC’s capabilities are predominantly situated at the lower levels of the cognitive hierarchy (eg, information retrieval and knowledge comprehension), with diminishing efficacy at higher levels requiring analysis, evaluation, and creation. This hierarchy effectively illustrates the inherent limitations of current AIGC tools in cultivating advanced critical thinking skills. Addressing these challenges necessitates a collaborative effort among educators, students, and policymakers to ensure that the integration of AIGC in medical education enhances, rather than impedes, the comprehensive development of students through appropriate guidance and effective regulation.

### Potential of AIGC in Fostering Critical Thinking

#### Student Perspectives

Numerous surveys indicate a nuanced and often contradictory perspective regarding AIGC. In a 2024 survey conducted by Abdullah et al [13], it was reported that 69% of medical students acknowledged the potential advantages of AIGC in fostering critical thinking, while 31% expressed concerns regarding its potential to induce mental inertia. A subsequent study by Moskovich and Rozani [14] in 2025 revealed a prevalent apprehension among medical students that AIGC may undermine critical thinking skills and promote complacency. Notably, a 2023 investigation by Almazrou et al [15] indicated that as many as 92.6% of medical students recognized the prospective benefits of AIGC in enhancing critical thinking. Furthermore, research by Abouammoh et al [16] suggested that students exhibited a more pragmatic outlook compared to educators, who tended to highlight “critical thinking deficits.”

These findings imply that while medical students generally perceive AIGC as a valuable tool for improving critical thinking, they simultaneously harbor concerns about the potential for mental inertia, thereby illustrating the dual opportunities and challenges presented by the integration of AIGC in medical education.

#### Standardized AI Patients

In recent years, the utilization of virtual standardized patients (SP) has emerged as an innovative tool in medical education, garnering increasing attention for its potential to enhance the critical thinking skills of medical students. A study conducted by Jiang et al [17] in 2024 revealed that the integration of virtual SP training with case-based learning significantly enhances students’ systematic and evidence-based thinking abilities. However, the study noted that the short-term effects on higher-order critical thinking skills were not as pronounced, indicating that a more profound integration of virtual SP training is necessary to effectively foster the development of critical thinking among medical students. Furthermore, in 2025, Öncü et al [18] highlighted the considerable advantages of AIGC, such as ChatGPT-4o, in terms of cost-effectiveness, accessibility, and standardization as a virtual standardized patient. This advancement presents new opportunities for the optimization and promotion of virtual SP training.

Nevertheless, from the perspective of critical thinking development, further investigation is required to explore how the benefits of AIGC can address the limitations of virtual SP training in enhancing higher-order thinking skills.

#### Customized AIGC Tools

In 2024, Shalong et al [19] conducted a randomized controlled trial that revealed the efficacy of a tailored GPT AI tool, which offers structured guidance rather than direct responses, in enhancing students’ scores on the Cornell Critical Thinking Test over a 12-week period and beyond. This finding indicates that the development of critical thinking skills among medical students can be effectively facilitated through the thoughtful design of AIGC guidance tools. In 2025, Reinke et al [20] introduced a framework for “guided use,” which positions AIGC as a critical thinking “partner” rather than a mere “substitute.” Additionally, Elabd et al [21] employed AIGC, such as ChatGPT and DALL-E 3, to create personalized multimodal memory methods tailored to various learning styles, while emphasizing the importance of treating these tools as supplementary resources to mitigate the risk of over-reliance. Furthermore, Wang et al [22] advocated for the development of open-ended tasks, such as clinical data analysis, and the establishment of case libraries showcasing AIGC applications (including examples of both accurate and inaccurate uses) within medical education, particularly in programming courses, to cultivate students’ critical utilization skills. Collectively, these studies and recommendations underscore the potential of customized AIGC tools in medical education to enhance critical thinking abilities and accommodate diverse student needs through personalized and varied learning approaches. However, the effectiveness of these tools is contingent upon their design and positioning to avert potential dependency risks. In contrast, Gong et al [23] examined the clinical applicability of customized LLMs in gastroenterology and found that the generic GPT-4o, equipped with retrieval-augmented generation capabilities, performed at an expert level, significantly surpassed the performance of customized models.

#### New Model of Resource Integration

Montagna et al [24] conducted a systematic comparison of the efficacy of 3 distinct types of clinical decision support systems: traditional clinical practice guidelines, online knowledge bases (such as UpToDate), and LLMs (ChatGPT) in the context of medical student case resolution. Their findings indicated that while ChatGPT provided the quickest responses, clinical practice guidelines demonstrated superior accuracy. Concurrently, Mondal [25] explored the potential influence of a novel model of resource integration on the critical thinking abilities of medical students. Their survey revealed that medical students frequently utilize AI, particularly LLMs, to rapidly construct knowledge frameworks, subsequently validating detailed information through authoritative databases, such as PubMed. This hybrid approach, termed “LLM (Large Language Model) + Search Engine + Authoritative Database,” not only facilitates the efficient integration of multisource information but also fosters the development of students’ “multiresource integration ability.” This capability is essential for addressing potential obsolescence or inaccuracies in AIGC-generated information, while also empowering students to maintain critical thinking when confronted with diverse information sources, thereby enhancing their skills in identifying and verifying the accuracy and reliability of information. This innovative model offers medical students a more holistic educational pathway, ultimately aiding in the enhancement of their critical thinking and decision-making competencies within complex informational contexts.

#### Identify Limitations and Proactively Assess

Laupichler et al [26] conducted a comparative analysis of the quality of questions generated by ChatGPT-3.5 versus those created artificially, revealing that AI-generated questions were more susceptible to being “guessed,” while artificially constructed questions were more effective in accurately assessing students’ true capabilities. Thomae et al [27] further illustrated the benefits of engaging students in the design of solutions and subsequent comparisons with AI-generated alternatives within the same academic year, particularly in the context of a “placebo control condition” unit. This comparative exercise not only enables students to visually discern the strengths and weaknesses of AI but also promotes critical thinking through facilitated discussions. Additionally, Mondal [28] emphasized the necessity for students to actively articulate their evaluations of AI-generated information and engage in discourse regarding its potential inaccuracies and biases. This practice is not only fundamental to the development of critical thinking skills but also essential for navigating the limitations of AI technology. Collectively, these studies underscore that through the processes of comparison, evaluation, and discussion of AI-generated content, medical students can enhance their critical thinking abilities, thereby enabling them to apply AI technology more judiciously in their future medical practices.

In summary, AIGC functions simultaneously as an enhancer and a potential inhibitor of critical thinking among medical students. When utilized appropriately, AIGC can evolve into an effective “practice partner.” However, improper use may lead to adverse outcomes, such as AIGC dependency syndrome, cognitive stagnation, and a deterioration of academic integrity. Addressing these challenges requires an integrated approach comprising standardized AIGC patient simulations, personalized instructional guidance, multifaceted evaluation methods, and proactive assessment strategies.

### Synopsis of Capabilities, Quick Check Applications, and Critical Thinking Hazards for Medical Students

This study systematically synthesized the dispersed findings from the 24 reviewed papers and developed a “risk-return matrix” (Table 4). The matrix is structured with the typical cognitive processes of medical students utilizing generative AIGC represented along the vertical axis: data input (dimensionality), information retrieval (application), standardization compliance (standardization), knowledge integration (knowledge), and deep clinical reasoning (deep thinking). The horizontal axis delineates the appealing “quick-check” functionalities available at each stage, including second-level differential diagnosis, automated extraction of key literature points, and collaborative draft decision-making between physicians and patients.

**Table 4.** Application of artificial intelligence–generated content (AIGC) to the critical thinking of medical students.

Dimensionality	Clinical diagnosis	Evidence-based medicine	Ethical decision-making	The capacity for scientific research
Application scenarios	Enter virtual patient symptoms, practice history acquisition, and differential diagnosis AI^a^ inquiry synchronous prompt differential diagnosis, reduce missed diagnosis	Quick search of the latest literature and treatment guidelines Quick search assisted retrieval of textbook standardization knowledge Quick Check Scholar AI plugin to extract key points from paper points	Assisting doctor-patient joint decision making	Assisted thesis writing (AI contribution needs to be marked transparently)
Advantages of rapid inspection technology	Rapid detection reduces the likelihood of missed diagnosis	Rapid inspection provides the latest evidence-based evidence Rapid Search to improve knowledge retrieval efficiency	A proposal for a standardized ethical decision framework for rapid inspection	Quick reference generation (accuracy needs to be verified)
Rapid detection of potential risks	Rapid inspection—students rely too much on AI "quick solutions," weakening deep thinking ability Rapid detection—lack of practical experience in differential diagnosis, resulting in insufficient emergency response capability Quick detection—AI generates error messages (eg, "hallucination" problem) Quick check—unprofessional questions lead to misleading answers	Quick check—lack of references to trace sources Quick check—answers may be oversimplified or knowledge fragmented Quick check—authoritative intonation covers errors	Quick checks risk oversimplification; they promote solutions that are morally idealistic but practically unworkable Rapid detection—social biases (race, gender) in training data are reinforced Quick check—Patient Privacy breach (HIPAA^b^ or GDPR^c^ violations)	Quick detection—generating false or erroneous references Quick detection—promoting plagiarism Quick check—marginal medical views are overwritten by mainstream content
Quick check deep challenge	Rapid examination—LLM^d^ fails to mimic human doctors’ comprehensive reasoning abilities Quick examination—unprofessional questioning by students leads to misdiagnosis risk	Quick check—relying on open access databases, information may not be comprehensive Challenge: Nonexperts’ difficulty in verifying fast-generated outputs	Rapid inspection—difficulty in clarifying AI’s responsibility boundaries in medicine Fast check data entry compliance issues	Fast detection—initial data bias is reinforced by loop learning Quick inspection-expert cross-validation offsets efficiency gains

a AI: artificial intelligence.

b HIPAA: Health Insurance Portability and Accountability Act.

c GDPR: General Data Protection Regulation.

d LLM: large language model.

Adjacent to each function on the right side, the matrix presents the “rapid-detection” outcomes derived from the systematic review. This comparative framework enables readers to promptly discern the specific contexts in which AIGC effectively enhances efficiency, as well as identify the stages where it may inadvertently undermine the critical thinking and clinical judgment skills of medical students.

Table 4 delineates the specific application scenarios of AIGC across various dimensions, including data input, information retrieval, knowledge integration, and deep reasoning. Examples encompass virtual patient symptom input, rapid literature retrieval, auxiliary differential diagnosis, and support in academic writing. Concurrently, the table highlights potential risks associated with these applications, such as students’ overreliance on AI-generated quick responses, which may contribute to cognitive decline, diminished capacity for deep analytical thinking, and challenges arising from errors or biases inherent in AIGC-produced content. The discussion underscores the necessity for the judicious use of AIGC within medical education, urging educators and learners to balance efficiency gains with vigilance regarding the possible adverse effects on the cultivation of medical students’ critical thinking skills and professional competencies.

## Discussion

### Principal Findings

The integration of AIGC technology into medical education exhibits a dual nature. As outlined in Table 4, which summarizes the 4 dimensions of critical thinking as applied by AIGC to medical students, this technology offers substantial benefits. On the one hand, AIGC enhances the educational experience by providing an abundance of learning resources and efficient tools, leveraging its advanced capabilities in information generation and processing. This facilitates a more rapid acquisition of knowledge and expands the scope of learning for medical students. Conversely, excessive reliance on AIGC may lead to cognitive inertia, a decline in critical thinking skills, and potential issues related to academic integrity. Nevertheless, through the strategic optimization of AIGC tool design and implementation, alongside appropriate guidance and oversight from educators, AIGC can serve as a valuable asset in fostering critical thinking among medical students. The research indicates that approaches such as the development of customized AIGC tools, the use of virtual standardized patients, innovative models for resource integration, and proactive assessments of AIGC limitations can effectively address the deficiencies of AIGC in cultivating advanced critical thinking skills. These strategies can assist medical students in sustaining their critical thinking abilities throughout the learning process, thereby strengthening their analytical and problem-solving capabilities.

### Limitations

While we made every effort to comprehensively retrieve relevant literature using the PRISMA framework, our research predominantly centered on ChatGPT’s applications. Future research should expand to other generative AI models to holistically evaluate their potential and differences in medical education. Currently, there is a scarcity of comparative studies on different models within the academic community, which restricts the in-depth analysis of their similarities and differences. To address this, future research could employ cross-model comparative experiments to explore each model’s unique advantages in cultivating critical thinking.

The study systematically reviewed literature to examine how AIGC technology affects medical students’ critical thinking and proposed coping strategy frameworks. However, several limitations might affect the findings’ comprehensiveness and the conclusions’ generalizability. The literature search was limited to studies published between November 2022 and June 2025. Although this captures recent AIGC advancements in medical education, it may miss earlier valuable research and post-June 2025 studies that could reveal significant trends.

Despite evaluation by 2 independent reviewers to reduce bias, some subjectivity may still exist in assessing studies’ relevance and quality. While disagreements were resolved through discussion, they could still somewhat influence the objectivity. The reviewed studies showed marked variability in design, sample size, participant characteristics, and assessment tools. Although this diversity enriches the research, it also causes result heterogeneity, compromising conclusion consistency and generalizability.

As AIGC technology rapidly evolves, the referenced literature and case studies may not fully reflect the latest developments. While AIGC excels in knowledge generation and information retrieval, concerns remain about the accuracy and reliability of its content. AI models might be limited by training data biases, algorithmic limitations, and language comprehension challenges, risking the generation of incorrect or misleading information. This could negatively impact medical students’ critical thinking, yet research on this issue is still inadequate.

Moreover, this study mainly focused on AIGC’s short-term effects on medical students’ critical thinking. As critical thinking development is a long-term process, further research is needed to investigate AIGC’s long-term effects, such as its potential to cause cognitive inertia or dependency. Additionally, due to potential methodological limitations in some included studies (eg, small sample sizes and lack of control groups), the study’s conclusions require further verification based on higher-quality evidence.

### Outlook

The future design of AIGC tools should prioritize customization and personalization tailored to the learning styles, knowledge levels, and educational needs of medical students. This approach entails the provision of targeted learning resources and guidance methods. By utilizing intelligent recommendation systems and adaptive learning platforms, AIGC can assist students in enhancing their critical thinking skills. Medical students are encouraged to implement a hybrid learning strategy that integrates LLMs, search engines, and authoritative databases. This strategy fosters the ability to critically evaluate diverse information sources and synthesize multiple resources effectively. Such competencies not only improve students’ information literacy but also bolster their decision-making capabilities in complex informational contexts. Furthermore, medical education should emphasize the development of AI literacy among students, enabling them to critically assess the advantages and limitations of AIGC technology and use AI tools judiciously in clinical practice. Through education in AI ethics and critical thinking, students can cultivate appropriate technical concepts and professional attributes.

## Supplementary material

10.2196/79939 Checklist 1 PRISMA-LSR checklist.

## Abbreviations

AI: artificial intelligence
AIGC: artificial intelligence–generated content
LLM: large language model
PRISMA: Preferred Reporting Items for Systematic Reviews and Meta-Analyses
SP: standardized patient
